# Mixing Effects of Understory Plant Litter on Decomposition and Nutrient Release of Tree Litter in Two Plantations in Northeast China

**DOI:** 10.1371/journal.pone.0076334

**Published:** 2013-10-15

**Authors:** Lei Zhao, Ya-Lin Hu, Gui-Gang Lin, Yong-chao Gao, Yun-Ting Fang, De-Hui Zeng

**Affiliations:** 1 State Key Laboratory of Forest and Soil Ecology, Institute of Applied Ecology, Chinese Academy of Sciences, Shenyang, Liaoning, China; 2 University of Chinese Academy of Sciences, Beijing, China; 3 Daqinggou Ecological Station, Institute of Applied Ecology, Chinese Academy of Sciences, Shenyang, Liaoning, China; DOE Pacific Northwest National Laboratory, United States of America

## Abstract

Understory vegetation plays a crucial role in carbon and nutrient cycling in forest ecosystems; however, it is not clear how understory species affect tree litter decomposition and nutrient dynamics. In this study, we examined the impacts of understory litter on the decomposition and nutrient release of tree litter both in a pine (*Pinus sylvestris* var. *mongolica*) and a poplar (*Populus* × *xiaozhuanica*) plantation in Northeast China. Leaf litter of tree species, and senesced aboveground materials from two dominant understory species, *Artemisia scoparia* and *Setaria viridis* in the pine stand and *Elymus villifer* and *A. sieversiana* in the poplar stand, were collected. Mass loss and N and P fluxes of single-species litter and three-species mixtures in each of the two forests were quantified. Data from single-species litterbags were used to generate predicted mass loss and N and P fluxes for the mixed-species litterbags. In the mixture from the pine stand, the observed mass loss and N release did not differ from the predicted value, whereas the observed P release was greater than the predicted value. However, the presence of understory litter decelerated the mass loss and did not affect N and P releases from the pine litter. In the poplar stand, litter mixture presented a positive non-additive effect on litter mass loss and P release, but an addition effect on N release. The presence of understory species accelerated only N release of poplar litter. Moreover, the responses of mass loss and N and P releases of understory litter in the mixtures varied with species in both pine and poplar plantations. Our results suggest that the effects of understory species on tree litter decomposition vary with tree species, and also highlight the importance of understory species in litter decomposition and nutrient cycles in forest ecosystems.

## Introduction

Litter decomposition is one of the key processes in terrestrial ecosystems, affecting nutrient availability and the carbon (C) cycle [1], [2]. Generally, litter decomposition rates and nutrient dynamics are influenced by abiotic and biotic factors, such as climate, litter quality, and the composition of the decomposer community [3], [4]. Most previous studies, however, have only focused on single-species litter decomposition rates and nutrient release. In both natural and artificial ecosystems, plant litter does not decompose alone; it usually decomposes together with co-existing plant species [5], [6].

Litter diversity can influence litter decomposition rates and nutrient dynamics, either by generating non-additive effects (synergistic or antagonistic) or additive effects [7], [8]. According to a literature review by Gartner and Cardon [9], more than two-thirds of experimental cases on mixture decomposition show non-additive effects on mass loss and nitrogen (N) release. There are three possible mechanisms to explain litter mixture effects on decomposition [8], [10], [11]. First, active microbial transfer via fungal hyphae and/or passive diffusion via leaching of nutrients can promote the decomposition of nutrient-poor litter [12]. Second, the release of some secondary metabolites such as tannins and polyphenols from some litter types can inhibit microbial activity and thus slow the decomposition of litter [13]. Third, a diverse-species litter layer can alter microclimatic conditions and microbial community composition and have indirect consequences for decomposition [14]. Hence, litter decomposition of single species does not sufficiently represent litter decomposition processes at an ecosystem level.

Understory vegetation, as an important component of forest ecosystems, plays a key role in maintaining ecosystem biodiversity, nutrient cycling, and productivity [15]–[17]. Decomposition rates and nutrient dynamics of tree litter may be affected by understory species litter via chemistry, morphology, moisture retention, and decomposer community differing from the tree litter at the floor-soil interface where litter decomposition occurs [18], [19]. Although many previous studies have examined the potential interaction effects of mixed litter on decomposition rates and nutrient dynamics [11], [20]–[22], these studies often focused on herbaceous litter mixtures or tree litter mixtures without consideration of the interactive effects between tree species and understory species on decomposition. Moreover, most of the previous studies usually measured mass loss and nutrient release of litter mixtures during decomposition as a whole to compare with the predicted values calculated on the basis of single-species litter decomposition. This did not differentiate the responses of individual species and examine their distinct roles in the mixture [10], [23]. Such poor understanding of the interaction of trees and their coexisting understory species has hindered the prediction of relationships between species diversity and ecosystem functioning in litter decomposition in forest ecosystems.

In most of the experimental studies on litter decomposition, the chemical composition (e.g. nutrient concentration, C/N ratio or lignin/N ratio) of litter is frequently considered to indicate its quality as a resource for decomposing organisms [24], and is often the determinant of litter decomposition in a given physical environment [25]. Nutrient-rich leaves decompose rapidly because they have high concentrations of labile compounds such as proteins and low concentrations of recalcitrant cell-wall components such as lignin [26]. Usually, deciduous tree leaves have higher nutrient concentrations and lower lignin concentrations than evergreen tree leaves, allowing deciduous tree leaves to decompose more rapidly than evergreen tree leaves [27]. Muller [28] found that, on average, decomposition rates of herbaceous litter were twice that of tree litter due to higher nutrient concentrations in the herbaceous litter, which facilitated nutrient cycling in forests.

In this study, we performed an experiment on mixed-litter decomposition in a Mongolian pine (*Pinus sylvestris* var. *mongolica*) and a Xiaozhuan poplar (*Populus* × *xiaozhuanica*) plantation, which are two major afforestation types for wind-erosion control in semi-arid regions of northern China [29]. We separately analyzed litter mass loss and nutrient dynamics of each species in litter mixtures from both pine and poplar plantations and examined the mixing effects of tree and understory litter during decomposition, which might be more conducive to the understanding of species interaction on litter decomposition. We proposed that the mixing effects on decomposition of tree litter and understory litter may depend on initial litter quality and that the mixing with high quality litter will stimulate the decomposition of low quality litter. Conversely, the mixing effect will not be significant when the litter quality is similar among the tree and understory species. Specifically, we expected that: (1) in the pine stand, the mass loss and nutrient release of pine litter mixed with understory litter would be accelerated compared with pine litter decomposing alone, and (2) in the poplar stand, the accelerated effects on poplar litter decomposition would not be significant since poplar and understory litter may be close in quality.

## Materials and Methods

### Site description

This study was conducted in a Mongolian pine and a Xiaozhuan poplar stand in the Daqinggou Ecological Station (42°58′ N, 122°21′ E, 260 m above sea level), located in southeastern Keerqin Sandy Lands, Northeast China. This area is located in a temperate climatic zone. The mean annual temperature is 6.4°C, with the lowest mean monthly temperature in January (–12.5°C) and the highest in July (23.8°C). The mean annual precipitation is 450 mm, with more than 60% occurring in June–August, and the mean annual frost-free period is approximately 150 days. The soil is a sandy soil (Typic Ustipsamment) that developed from eolian parent material and the textural composition is 90.9% sand, 5.0% silt, and 4.1% clay. The soil organic C, total N and total P concentrations are 3.15, 0.24 and 0.09 g kg^−1^, respectively [29].

### Litter preparation and experimental design

In October 2010, fresh leaf litter of pine and poplar was collected from an 11 year-old pine plantation and a 20 year-old poplar plantation, respectively. The stand density is about 1000 and 1800 trees ha^−1^ in the pine and poplar plantations, respectively. *Artemisia scoparia* and *Setaria viridis* were the dominant herbs under the pine plantation; *Elymus villifer* and *A. sieversiana* were the dominant herbs under the poplar plantation. We clipped herbaceous litter from the ground and cut into 5-cm-long sections. Samples of air-dried litter (4 g) were enclosed in 10 cm ×10 cm nylon bags with a top layer of 2 mm nylon mesh, and a bottom layer of 1 mm nylon mesh. These two mesh sizes were chosen to avoid litter escaping and to allow for soil fauna entrance [30]. The mass proportion of tree litter and its understory litter in mixture was 50:35:15, i.e., pine, *A. scoparia* and *S. viridis* occupied 50%, 35% and 15% in the pine plantation, and poplar, *E. villifer* and *A. sieversiana* occupied 50%, 35% and 15% in the polar plantation, respectively. The mixing proportion was defined according to their relative biomass of aboveground litter in each of the two forest stands. Subsamples of litter were oven-dried at 65°C to develop conversion factors from air-dry mass to oven-dry mass. In our incubation experiment, four treatments were installed for each stand: three with litterbags containing litter of an individual species and one with a three-species mixture. For the mixed-species litterbags, individual components were uniformly mixed. Litterbags were placed in direct contact with soil under their corresponding Mongolian pine and poplar plantations on November 10, 2010. In total, 96 litterbags were used (four treatments × four replications × three times sampling × two stands).

### Sampling and chemical analysis

Four litterbags were retrieved from each treatment after 5, 9, and 12 months of field incubation. Litter was removed from each litterbag, oven-dried (65°C) for 48 h, and weighed to determine the percentage to the original mass. For the mixed-litter treatments, each component species was separated from the mixtures on the basis of morphology.

Oven-dried litter was ground to a fine powder and passed through a 0.2 mm sieve for chemical analysis. Total C concentration was measured by wet oxidation with potassium dichromate [31]. Total N concentration was measured by the Kjeldahl method [32] and total P concentration was measured by the molybdenum-stibium colorimetry method with a continuous-flow autoanalyzer (AutoAnalyzer III, Bran + Luebbe GmbH, Germany) after the samples were digested with H_2_SO_4_. We measured lignin using a modified acetyl bromide method [33] with samples calibrated against a standard of lignin (lignin, alkali, 2-hydroxypropyl ether). Briefly, for each litter sample, a 6-mg subsample was placed in a 50 ml graduated tube with a solution of 25% acetyl bromide (AcBr) in acetic acid (5 ml) and added 0.2 ml HClO_4_ (70%). The tube was sealed with a cap and placed in an oven at 70±0.2°C for 30 minutes. The tube was shaken at 10-min intervals to promote dissolution of the sample. Then, 10 ml 2 mol L^−1^ NaOH was added and the solution was made up to 50 ml by adding acetic acid. The lignin content in the solution was measured by UV spectrophotometry at 280 nm.

### Data processing and statistical analysis

Observed nutrient remaining was calculated by the change of nutrient content during litter decomposition:
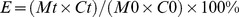
where *E* is the percentage of remained nutrient content to the initial value (%), *M*_0_ is the initial oven-dry mass (g), *C*_0_ is the initial nutrient concentration (mg g^−1^), *M_t_* is the oven-dry mass at time *t*, and *C_t_* is the nutrient concentration at time *t*.

For litter mixtures, the predicted remaining values of litter mass and nutrient content were calculated by the following equation [10]:
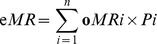
where e*MR* is the predicted value (%), o*MR_i_* is the observed value (%) of litter *i* decomposing alone and *P_i_* is the initial proportion of litter *i* in mixture.

One-way analysis of variance (ANOVA) was used to test the differences in initial litter chemical characteristics among species. Bivariate correlations were performed to examine the relationship between initial litter characteristics, mass loss, and nutrient remaining. The differences between the observed and predicted values of litter mass loss and N and P remaining in mixtures from each of the two stands, and the differences of the observed litter mass loss and N and P remaining values for each species between mixed- and single-species litterbags, at the end of incubation were tested using a paired *t*-test [34], [35]. A significant difference between the observed and predicted values indicates a non-additive effect, while no difference indicates an additive effect. Percentage data were log-transformed to satisfy the assumption of normality. All statistical analyses were performed using SPSS 13.0 for Windows Statistical Software package. The level of significance for statistical tests is *α* = 0.05.

## Results

### Initial litter chemistry

Initial litter chemistry varied dramatically with species (Table 1). Total C concentration of pine litter was significantly higher than that of the other species litter, with poplar having the lowest. In contrast, total N concentration of poplar litter was the highest, and pine litter had the lowest N concentration. Total P concentration was the highest in *A. sieversiana* litter, and was the lowest in pine litter. Lignin concentration of *A. scoparia* litter was higher than that of the other species litter, with the lowest being the poplar litter. Pine had higher litter C/N and lignin/N ratios than did its two dominant understory species. On the contrary, the C/N and lignin/N ratios of poplar litter were significantly lower than those of understory litter in the poplar stand (Table 1). Thus, in the pine stand, the litter quality of pine was lower than that of its corresponding two understory species (*A. scoparia* and *S. viridis*), while the converse was true in the poplar stand with the poplar having higher quality of litter compared to the two understory species (*E. villifer* and *A. sieversiana*, Table 1).

**Table 1:** Initial litter chemistry of *Pinus sylvestris* var. *mongolica* and *Populus* × *xiaozhuanica* and their dominant understory species.

Stand	Litter species	Total C (mg g^−1^)	Total N (mg g^−1^)	Total P (mg g^−1^)	Lignin (mg g^−1^)	C/N	Lignin/N
Pine	Psy	551 (7) a	3.6 (0.1) d	0.23 (0.01) e	306 (8) b	153 (5) a	85 (1) a
	Asc	473 (7) b	12.4 (1.4) b	2.30 (0.22) a	340 (10) a	38 (5) cd	27 (3) c
	Svi	431 (3) d	4.2 (0.4) d	1.24 (0.01) b	267 (15) c	103 (9) b	64 (7) b
Poplar	Pxi	413 (5) e	15.3 (0.7) a	0.94 (0.01) c	235 (8) d	27 (1) d	15 (1) d
	Evi	454 (5) c	10.0 (0.9) c	0.63 (0.02) d	305 (3) b	45 (4) c	31 (3) c
	Asi	458 (7) c	10.9 (1.2) bc	1.18 (0.18) bc	294 (7) b	42 (5) c	27 (3) c

Psy, *Pinus sylvestris* var. *mongolica*; Asc, *Artemisia scoparia*; Svi, *Setaria viridis*; Pxi, *Populus* × *xiaozhuanica*; Evi, *Elymus villifer*; Asi, *A. sieversiana*. Values are means with SD (*n* = 4). Different letters within the same column indicate significant differences at *P*<0.05.

### Mass loss

**Figure 1:**
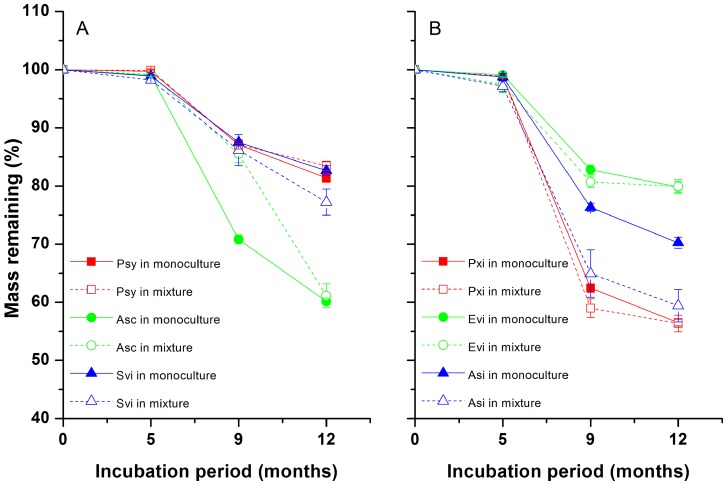
Litter mass dynamics in monocultures and mixtures of *Pinus sylvestris* var. *mongolica* (A) and *Populus* × *xiaozhuanica* (B) stands during a 12-month period of incubation. Values are means with SD (*n* = 4). Psy, *Pinus sylvestris* var. *mongolica*; Asc, *Artemisia scoparia*; Svi, *Setaria viridis*; Pxi, *Populus* × *xiaozhuanica*; Evi, *Elymus villifer*; Asi, *A. sieversiana*.

**Figure 2:**
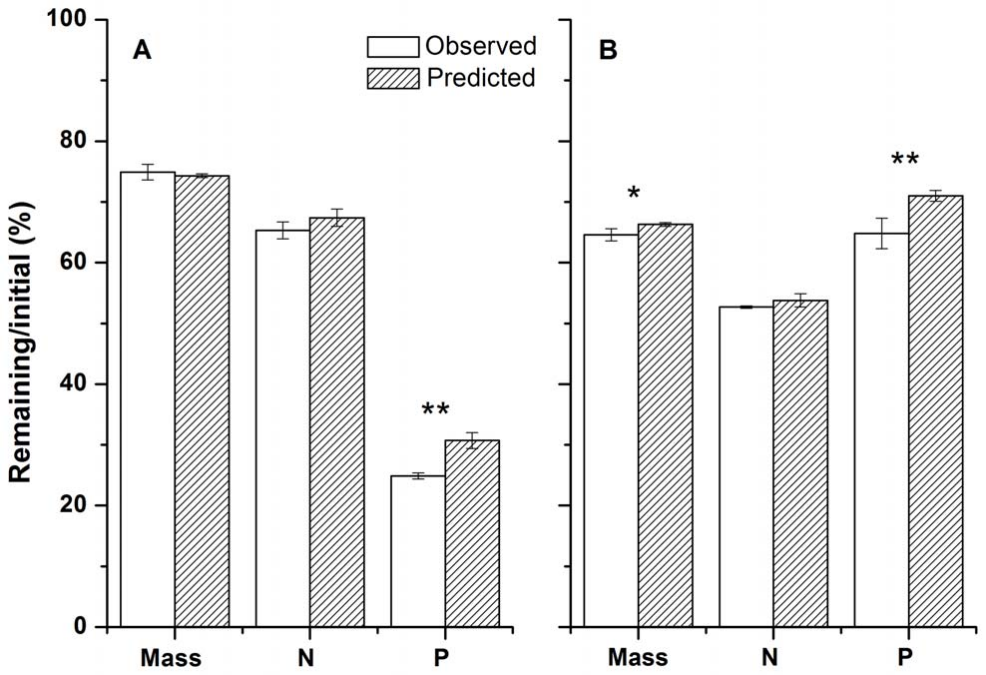
Observed and predicted litter mass, N and P remaining of mixtures in both *Pinus sylvestris* var. *mongolica* (A) and *Populus* × *xiaozhuanica* (B) stands after a 12-month period of incubation. The predicted values in mixtures were based on the decomposition of component species decaying alone. Values are means with SD (*n* = 4). * *P*<0.05, ** *P*<0.01.

In the Mongolian pine stand, all the three species in both the single-species and mixed-species litterbags had very low levels of litter mass loss during the first five months of incubation (in the winter from November to next April). Afterwards, they decomposed faster during the fifth to ninth months (from April to August; Fig. 1A). After one year of decomposition, pine litter exhibited lower mass loss in the mixture than in the monoculture (83.5% vs. 81.4% mass remaining, respectively; *P*<0.01; Table 2); however, *S. viridis* litter had significantly greater mass loss in the mixture than in the monoculture (77.2% vs. 82.6% mass remaining, respectively; *P*<0.05; Table 2). *A. scoparia* litter showed no change in mass loss in the mixture compared to that in the monoculture. Overall, there was no difference in total mass loss of the mixed litter in the pine stand between the observed and predicted values (*P* = 0.47, Fig. 2A).

**Table 2:** The significance of difference in remaining values of mass, N and P relative to initial values of each species litter between in monocultures and in mixtures after 12-*t* test (*P* values).

Variables	Stand	Litter species	Monoculture (%)	Mixture (%)	*P* value
Mass remaining	Pine	Psy	81.4 (0.7)	83.5 (0.8)	0.007
		Asc	60.1 (0.5)	61.1 (2.0)	0.484
		Svi	82.6 (0.9)	77.2 (2.2)	0.010
	Poplar	Pxi	56.5 (0.6)	56.4 (1.4)	0.856
		Evi	79.8 (0.9)	79.9 (1.2)	0.841
		Asi	70.2 (1.0)	59.4 (2.8)	0.004
N remaining	Pine	Psy	106.5 (2.2)	107.1 (2.2)	0.801
		Asc	51.3 (1.8)	44.3 (2.9)	0.022
		Svi	68.0 (2.5)	86.6 (2.4)	<0.001
	Poplar	Pxi	41.1 (0.9)	36.9 (1.2)	0.004
		Evi	82.9 (2.1)	82.3 (1.7)	0.770
		Asi	51.4 (0.7)	63.5 (3.0)	0.007
P remaining	Pine	Psy	93.2 (6.8)	96.7 (2.2)	0.361
		Asc	24.6 (1.9)	15.1 (1.4)	0.003
		Svi	22.1 (2.1)	22.6 (2.2)	0.695
	Poplar	Pxi	66.0 (1.0)	61.9 (3.1)	0.087
		Evi	81.2 (1.7)	84.3 (3.7)	0.257
		Asi	69.5 (6.3)	46.2 (2.5)	0.009

Psy, *Pinus sylvestris* var. *mongolica*; Asc, *Artemisia scoparia*; Svi, *Setaria viridis*; Pxi, *Populus* × *xiaozhuanica*; Evi, *Elymus villifer*; Asi, *A. sieversiana*. Values are means with SD (n = 4).

In the poplar stand, the litter mass loss of the three species in both the monoculture and the mixture showed similar temporal patterns to the Mongolian pine stand (Fig. 1B). Over one year, the mass loss of *A. sieversiana* litter was significantly accelerated in the mixture as compared to that in the monoculture (59.4% vs. 70.2% mass remaining, respectively; *P*<0.01; Table 2). The mass loss of poplar and *E. villifer* litter did not significantly change in the mixture as compared to that of the monoculture (*P* = 0.86 and *P* = 0.84, respectively). Thus, the observed total mass loss of mixed litter was significantly greater than the predicted value (*P* = 0.03, Fig. 2B).

### Nutrient release

**Figure 3:**
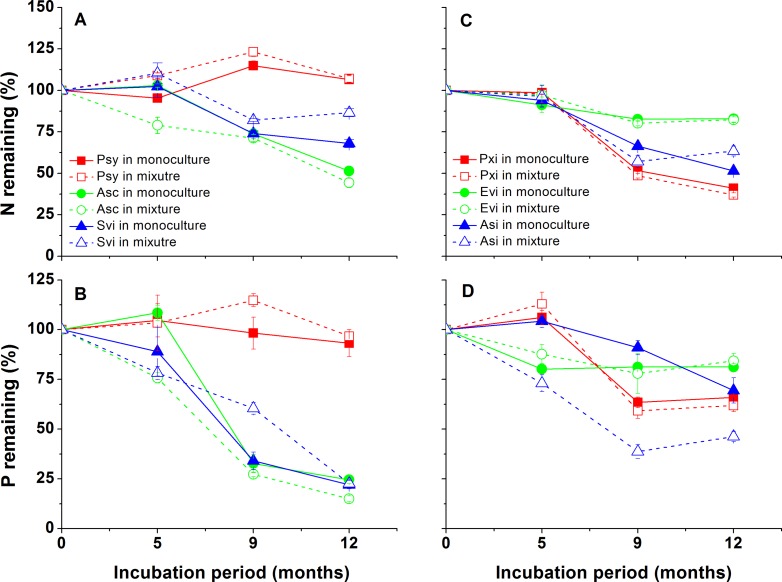
Litter N and P content dynamics in monocultures and mixtures of *Pinus sylvestris* var. *mongolica* (A and B) and *Populus* × *xiaozhuanica* (C and D) stands during a 12-month period of incubation. Values are means with SD (*n* = 4). Psy, *Pinus sylvestris* var. *mongolica*; Asc, *Artemisia scoparia*; Svi, *Setaria viridis*; Pxi, *Populus* × *xiaozhuanica*; Evi, *Elymus villifer*; Asi, *A. sieversiana*.

The contents of litter N and P nutrients changed in relation to the decomposition stage and litter type (Fig. 3). In the Mongolian pine stand, a slight N release occurred in pine litter in the monoculture at the initial 5 months of decomposition, compared to a slight N immobilization in the mixture. Afterwards, pine litter exhibited N immobilization (6.5–7.1%) as compared to its initial value in both the monoculture and the mixture at 12 months of incubation (Fig. 3A). Moreover, no difference in N remaining in the pine litter was observed after one year between the mixture and monoculture (Table 2). *A. scoparia* litter exhibited higher N release in the mixture than in the monoculture after one year (44.3% vs. 51.3% N remaining, respectively; *P*<0.05; Table 2), while *S. viridis* litter showed lower N release in mixture than in monoculture (86.6% vs. 68.0% N remaining, respectively; *P*<0.01; Table 2). With regard to P dynamics, pine litter showed little variation in P contents during the whole decomposition process irrespective of mixture or monoculture, with a slight release (3.3–6.8%) at 12 months as compared to its initial value (Fig. 3B). *A. scoparia* and *S. viridis* litter showed continuous P releases in both monoculture and mixture during the whole decomposition process, except for *A. scoparia* litter with a slight P immobilization in monoculture at 5 months (Fig. 3B). Ultimately, *A. scoparia* litter exhibited higher P release in the mixture than in the monoculture (15.1% vs. 24.6% P remaining, respectively; *P*<0.05; Table 2), while *S. viridis* litter showed no difference in P release between the mixture and the monoculture (22.6% vs. 22.1% P remaining, respectively; *P*>0.05; Table 2). Consequently, in the pine-dominant litter mixture, the observed value of total litter N remaining did not differ from the predicted value after 12 months of decomposition, while the observed value of total P remaining was lower than the predicted value (Fig. 2A).

In the poplar stand, all the three species exhibited N release in both the monoculture and the mixture during the whole decomposition process (Fig. 3C). After 12 months, the N release of poplar litter was significantly higher in the mixture than in the monoculture (36.9% vs. 41.1% N remaining, respectively; *P*<0.01; Table 2). The N release, however, of *A. sieversiana* litter was significantly lower in the mixture compared to the monoculture (63.5% vs. 51.4% N remaining, respectively; *P*<0.05; Table 2), while *E. villifer* litter showed no difference in N remaining between the mixture and the monoculture. Concerning P dynamics, poplar showed P immobilization at the initial 5-month period and substantial P release from the fifth to ninth months in both the monoculture and the mixture (Fig. 3D). *E. villifer* released P at the initial 5-month period and then P content remained almost constant in both the monoculture and the mixture. *A. sieversiana* litter in the monoculture immobilized P slightly at the initial 5-month period and released P in the later decomposition period, but in the mixture *A. sieversiana* showed P release until the ninth month and gradually immobilized P at the end of incubation. After 12 months of decomposition, no differences in P remaining in both poplar and *E. villifer* litter were observed between the monoculture and the mixture (Table 2). However, P remaining content of *A. sieversiana* litter was higher in the monoculture than in the mixture (69.5% vs. 46.2% P remaining, respectively; *P*<0.01; Table 2). Collectively, in poplar-dominant litter mixtures, the observed value of total litter N content did not differ from the predicted value after 12 months of decomposition, while the observed value of total litter P content was lower than the predicted value (Fig. 2B).

### Relationships between initial litter chemistry, mass loss, and nutrient release

Mass loss was positively correlated with the initial litter N and P concentrations, but negatively correlated with the C/N ratio and lignin/N ratio in both the monoculture and mixture treatments (Table 3). Similarly, the remaining N content was positively correlated with the initial N concentration, but negatively correlated with the C/N and lignin/N ratios. The remaining P content was positively correlated with the initial N concentration, but negatively correlated with the initial C concentration, C/N ratio and lignin/N ratio in both the monoculture and mixture treatments. N remaining showed a significant negative relationship with the initial C concentration in the mixture, however, showed no significant relationship with the initial C concentration in the monoculture. Moreover, P remaining showed no relationship with initial P concentration and a negative relationship with initial lignin concentration in the mixture, but showed opposite patterns in the monoculture.

**Table 3:** Pearson's correlation coefficients between initial litter quality variables and mass loss, N or P remaining contents of specific litter in monocultures and mixtures after 12 months of decomposition.

		Initial C	Initial N	Initial P	Initial lignin	Initial C/N	Initial lignin/N
Monoculture	Mass loss	−0.39 *ns*	0.88 **	0.54 **	−0.12 *ns*	−0.72 **	−0.75 **
	N remaining	−0.36 *ns*	0.90 **	0.29 *ns*	−0.09 *ns*	−0.84 **	−0.86 **
	P remaining	−0.48 *	0.86 **	0.45 *	−0.10 *ns*	−0.85 **	−0.87 **
Mixture	Mass loss	−0.50 *	0.79 **	0.56 **	−0.20 *ns*	−0.75 **	−0.77 **
	N remaining	−0.44 *	0.80 **	0.17 *ns*	−0.03 *ns*	−0.85 **	−0.87 **
	P remaining	−0.62 **	0.83 **	0.12 *ns*	−0.41 *	−0.81 **	−0.85 **

* *P*<0.5; ** *P*<0.01; *ns* not significant.

## Discussion

### Decomposition of pine litter mixed with understory litter

In this study, we found that in the pine stand, the litter mixture of Mongolian pine and its understory vegetation showed an additive effect on litter mass loss during decomposition with no significant difference between observed and predicted mass remaining (Fig. 2A). The result was not consistent with our first hypothesis. Wardle et al. [35] suggested that highly contrasting characters of litter mixed together do not necessarily affect the overall decomposition rate of the mixture. Blair et al. [36] also suggested that an additive effect of mixture decomposition might give a false impression that it was no mixing effect if two types of litter had opposite effects in the mixture decomposition (for instance, one was stimulated and the other was inhibited) and if the distinct roles of the individual species were not examined. Unlike many previous studies, we separately analyzed the mass loss and nutrient dynamics of each component species in the mixtures, which allowed us to examine the distinct responses of individual species during mixture decomposition. In the present study, we found that the decomposition of pine litter was inhibited while *S. viridis* litter decomposition was promoted in the litter mixture, which resulted in an additive effect on total mass loss of the litter mixture.

Similar to our results, Ganjegunte et al. [23] found that the decomposition rate of radiata pine litter mixed with its understory litter in laboratory microcosms was slower than that of pure radiata pine litter, and that the differences in chemistry of pure and mixed pine needle litter after 10 months of decomposition could explain the differences in mass loss. Litter decomposition rate is controlled by availability of nutrients and a readily available source of C [37], [38]. In mixed litter, a high initial N concentration coupled with greater concentrations of lignin in the litter will lead to the formation of highly stable lignin-protein complexes [23], which might have resulted in the reduced decomposition rate of the pine needle litter in our study. Moreover, N might be translocated from *A. scoparia* litter to *S. viridis* litter, which led to the stimulation of decomposition for *S. viridis* litter, but not for pine litter.

Many previous studies have reported that litter quality and decomposers are the controlling factors for decomposition within the same climate conditions [4], [39]. For the early stages of decomposition, the N concentration, C/N ratio, and lignin/N ratio are good predictors to assess litter decomposition rates and nutrient releases [40]–[43]. Hoorens et al. [44] found that initial litter C, P, and phenolic concentrations were correlated with decomposition rates, but not correlated with the non-additive effects of the mixture. However, Liu et al. [20] found that initial N and P concentrations of the litter not only strongly controlled decomposition rates, but also were significantly correlated with the non-additive effects of litter mixture. Indeed, we also found that litter mass loss was positively correlated with initial N and P concentrations and negatively correlated with C/N and lignin/N ratios.

Inconsistent with our hypothesis, the litter mixture of Mongolian pine and understory species had an additive effect on the N remaining (Fig. 2A). In addition, there was no difference in N remaining of pine litter between the monoculture and the mixture (Table 2). Hooper and Vitousek [45] suggest that increased litter species richness does not necessarily stimulate litter nutrient release. However, N remaining was decreased in the *A. scoparia* litter but increased in the *S. viridis* litter in the mixtures as compared to that of these two species decomposing alone, respectively. Such phenomena are consistent with the results of Ball et al. [46], who showed that tulip poplar and chestnut oak litter with higher N concentrations stimulated N release that was subsequently immobilized by the litter with lower N concentrations in mixed-litter decomposition. Gartner and Cardon [9] found that the N immobilization in a sugar maple and red oak litter mixture was lower than that predicted from the observed dynamics in single-species litterbags. Salamanca et al. [34] found that the N remaining content of *Pinus densiflora* litter was higher in a mixture than in a monoculture, while *Quercus serrata* had lower N remaining in a mixture than in a monoculture. The most likely reason is the translocation of nutrients from nutrient-rich litter to nutrient-poor litter.

The observed value of total litter P remaining was significantly lower than the predicted value in the Mongolian pine plantation (Fig. 2A), suggesting a synergistic effect on P release in the mixture. Polyakova and Billor [47] also found that P content of pine needles mixed with deciduous litter was lower than that of pure pine needles after approximately one year of decomposition. In our study, we did not observe a difference in P remaining in pine litter between the monoculture and the mixture. However, the P release of *A. scoparia* was greatly accelerated in the mixture as compared to that in the monoculture, which accounts for the positive non-additive effects on P release in the litter mixture.

### Decomposition of poplar litter mixed with understory litter

In contrast to the Mongolian pine stand, we found that there was a synergistic effect on mass loss after poplar litter was mixed with understory litter. This synergistic effect is consistent with several studies showing that litter mixtures with different chemical components decomposed faster than the predicted value [34], [48], [49]. The stimulated effect in the poplar stand was contributed to the increased decomposition rate of *A. sieversiana*, an understory species in the mixture (Table 2). However, the decomposition rate of poplar litter in the mixture did not change as compared to the monoculture, being consistent with our second hypothesis. No change in decomposition rate of the poplar litter may be due to its already low C/N ratio (Table 1).

Non-additive effects of litter mixtures may vary with litter chemical components [48], mixing ratios [6], [11], [27], and activities of decomposers [8], [10] during the decomposition process. *A. sieversiana* with a lower mass proportion (15%) in the mixture showed an accelerated mass loss although there were no obvious differences in C/N and lignin/N ratios between *A. sieversiana* and *E. villifer* (with a proportion of 35% in the mixture). This result suggests that a small proportion of litter could also have contributed to the occurrence of the non-additive effect in the mixture.

Although an additive effect on N release was found in the poplar-dominant litter mixtures, N release in poplar litter was accelerated in the mixture compared to the monoculture while the *A. sieversiana* showed a decreased N release in the mixture (Table 2). Microbial organisms preferentially exploit N nutrient released from higher-quality litter, whereas lower-quality litter immobilizes N and provides a resource for further decay [46]. In our study, the N-rich poplar litter would be easy to translocate N to the N-poor *A. sieversiana* litter. Moreover, the P release of *A. sieversiana* with higher initial P concentration was significantly greater in the mixture than in the monoculture, while the P release in both poplar litter and *E. villifer* litter was not significantly changed, which led to a positive non-additive effect on P release in the mixture.

## Conclusions

This study provides an opportunity to understand the relationships between biodiversity and ecosystem functioning from the viewpoint of litter decomposition, and confirms the important role of understory species in litter decomposition of forests. We initially hypothesized that the presence of understory species would stimulate the decomposition of pine leaf litter (low quality, with high C/N and lignin/N ratios) and have no effects on the high-quality poplar leaf litter. However, these hypotheses were not supported in the pine plantation; our data showed that the presence of understory litter inhibited the decomposition of Mongolian pine leaf litter. Our results suggest that the mixing effects of trees and their coexisting understory species in litter decomposition differ with tree species, depending on the initial litter chemical properties of the component species in the ecosystems. Our results also highlight that during decomposition, the interaction between tree species and understory species may regulate changes in litter chemistry, which could influence the functioning of litter-derived soil organic matter and the release of nutrients. Therefore, understory vegetation and its litter should be given more concerns in forest ecosystem management. Considering that much of the mass and N (and P in some cases) were still remaining in the litter (especially in the pine plantation) in our study after one year of decomposition, we recommend that longer-term studies will help quantify changes in mass loss and nutrient release between observed and predicted values in the latter stages of decomposition.
